# Fetal Long QT Syndrome: Case Series and Literature Review With Focus on Multidisciplinary Care Coordination

**DOI:** 10.1155/cric/5600959

**Published:** 2026-03-11

**Authors:** Stefani Samples, Sara Cherny, Stephanie Chandler, Ronald T. Wakai, Janette F. Strasburger, Sheetal Patel

**Affiliations:** ^1^ Pediatric Cardiology, Ann & Robert H. Lurie Children′s Hospital of Chicago, Chicago, Illinois, USA; ^2^ Department of Pediatrics, Feinberg School of Medicine, Northwestern University, Chicago, Illinois, USA, northwestern.edu; ^3^ Department of Medical Physics, University of Wisconsin–Madison, Madison, Wisconsin, USA, wisc.edu; ^4^ Pediatric Cardiology, Children′s Wisconsin–Milwaukee, Milwaukee, Wisconsin, USA

## Abstract

Congenital long QT syndrome (LQTS) is a group of heritable conditions that are associated with cardiac repolarization abnormality characterized by QT prolongation on ECG and risk of life‐threatening arrhythmias. Prenatal detection of LQTS presents many challenges for clinicians and a multidisciplinary approach is needed for optimal prenatal and postnatal management. We describe five cases of fetal diagnosis of LQTS with variable initial presentation, diagnostic strategies, and management approaches. A multidisciplinary team approach including fetal cardiologist, adult and pediatric electrophysiologists, medical physicists, neonatologists, maternal–fetal medicine specialists, fetal cardiac nurse coordinators, and genetic counselors allowed for comprehensive prenatal management and well‐planned postnatal treatment to optimize neonatal and maternal outcomes.

## 1. Introduction

Congenital long QT syndrome (LQTS) is a group of heritable conditions associated with a cardiac repolarization abnormality characterized by QT prolongation on electrocardiogram (ECG) and risk of life‐threatening arrhythmias. Prenatal detection of LQTS presents challenges for clinicians. We describe five cases of fetal diagnosis of LQTS with variable presentation, diagnostic strategies, and management approaches to highlight disease complexity and the importance of a multidisciplinary approach for optimal prenatal and postnatal management.

## 2. Case Series

Here, we present five distinct cases of fetal LQTS, including outcomes, for comparison and contrast (Table 1). Each had a unique fetal presentation, prenatal diagnostic evaluation, family history, genetics, and individualized management plan. A multidisciplinary team including fetal cardiologist, adult and pediatric electrophysiologists, medical physicists, neonatologists, maternal–fetal medicine (MFM) specialists, fetal cardiac nurse coordinators, and genetic counselors was used in each case.

**Table 1:** Full details for five cases of fetal Long QT syndrome for side by side comparison.

Case	1	2	3	4	5
**Maternal info**	34‐year‐old G2P1	29‐year‐old G2P1	34‐year‐old G1P0	32‐year‐old G2P0	36‐year‐old G4P1
**EGA at referral (weeks)**	29.3	32.4	30.2	21.1	21.6
**Reason for referral**	Abnormal fetal heart rhythm	Concern for fetal arrhythmia	Concern for tapered aortic arch	Fetal bradycardia	Maternal history of LQTS
**Maternal past medical history**	LQTS Type 2 diagnosed after TdP (subsequent positive genetic testing), s/p ICD placement	Unremarkable	Unremarkable	Unremarkable	LQTS Type 1 (positive genetic testing)
**Maternal medications**	Nadolol 10 mg qday	N/A	N/A	N/A	Propranolol 10 mg BID
**Family history**	Previous child with LQTS, multiple maternal relatives with LQTS	None	None	Paternal aunt with LQTS (*KCNQ1* +). Father with prolonged QTc on ECG (no genetic testing)	Father, two sisters, and four nieces/nephews with known LQTS Type 1 (same genetic mutation)
**Workup**	Fetal echo, fMCG, and maternal antibody testing	Fetal echo, fMCG, maternal ECG, and maternal antibody testing	Fetal echo, maternal ECG, and fMCG	Fetal echo, maternal genetic testing, and fMCG	Fetal echo and fetal genetic testing
**Fetal echo results**	Fetal bradycardia with 2:1 AV block (Figure 1)	High‐degree AV block, abnormal myocardium, and severe biventricular systolic dysfunction	Fetal bradycardia (115–124 bpm) with normal AV interval; IVRT: 58–66 ms	Fetal bradycardia (119–126 bpm) with 1:1 AV conduction; IVRT: 60–76 ms	Mild sinus bradycardia (127–138 bpm) with normal AV interval; IVRT: 56–62 ms
**fMCG Results**	Prolonged QTc 550 ms with occasional 2:1 AV conduction	Prolonged QTc (> 600 ms), periods of TdP at 200 bpm, predominantly 2:1 and 3:1 AV block (Figure 2)	Prolonged QTc 517 ms with bradycardia (110–119 bpm)	Prolonged QTc 536 ms with bradycardia (112–119 bpm)	N/A
**Other testing results**	Maternal antibody testing: SSA/SSB negative	Maternal ECG: normal Maternal antibody testing: SSA/SSB negative	Maternal ECG × 3: borderline or prolonged QTc	Maternal genetic testing (see below)	Fetal genetic testing (see below)
**Fetal diagnosis**	LQTS Type 2	Presumed LQTS	Concern for LQTS	Presumed LQTS	LQTS Type 1
**Prenatal treatment**	Vitamin D, Magnesium, Nadolol 10 mg qday	Propranolol XL 120 mg qAM and 60 mg qPM, magnesium	N/A	Propranolol 120 mg qday, vitamin D	Propranolol 10 mg BID
**Fetal/neonatal genetics**	*KCNH2* *c*.1935*G* > *A* *(p.Met645Ile)*	*KCNH2* *c*.1898*A* > *G* *(p.Asn633Ser)*	*CALM2* *c*.272*G* > *T* *(p.Arg91Leu)* VUS *AKAP9 c.10273_10275del(p.Gln3425del)* VUS	*KCNQ1* *c*.1615*C* > *T* *(p.Arg539Trp)*	*KCNQ1* *c*914*G* > *A* *(pTrp305* ∗ *)*
**Familial genetics**	Maternal: *KCNH2* *c*.1935*G* > *A* *(p.Met645Ile)* variant	Maternal and paternal genetic testing negative	Maternal *CALM2* *c*.272*G* > *T* *(p.Arg91Leu)* VUS *AKAP9 c.10273_10275del (p.Gln3425del)* VUS	Maternal: pathogenic *KCNQ1 (* *c*.1615*C* > *T*) variant	Maternal: *K* *C* *N* *Q*1*c*914*G* > *A* *(pTrp305* ∗)
**Inheritance**	AD	De novo	AD	AD	AD
**Pregnancy Outcome**	38.2 week C/S delivery	Prenatal conversion to NSR; 38.6‐week SVD	37.1‐week NSVD	39.3‐week NSVD	39.2‐week SVD
**Postnatal ECG**	DOL 1: NSR, markedly prolonged QTc (574 ms) 2 months: atrial‐sensed, ventricular paced rhythm, prolonged QTc (518 ms) 1 year: atrial‐sensed, ventricular‐paced rhythm, prolonged QTc (514 ms)	DOL 1: sinus bradycardia, markedly prolonged QTc (605 ms) 2 months: NSR, prolonged QTc (530 ms) 1 year: sinus bradycardia, prolonged QTc (488 ms), abnormal T‐wave morphology	DOL 1: sinus bradycardia, prolonged QTc (503 ms) 6 months: NSR, prolonged QTc (477 ms)	DOL 1: prolonged QTc (561 ms) 6 months: sinus bradycardia, prolonged QTc (489 ms)	DOL 1: sinus bradycardia, prolonged QTc (545 ms) with abnormal T‐wave morphology 3 months: NSR, prolonged QTc (461 ms) with abnormal T‐wave morphology
**Pacemaker**	Placed on DOL 10 due to bradycardia and insufficient cardiac output	N/A	N/A	N/A	N/A
**Discharge medications**	Propranolol, mexiletine	Propranolol, mexiletine	Propranolol	Propranolol	Propranolol
**Postnatal diagnosis**	LQTS Type 2	LQTS Type 2	LQTS (familial VUS)	LQTS Type 1	LQTS Type 1
**Current status**	Outpatient follow‐up at 36 months	Outpatient follow‐up at 31 months	Outpatient follow‐up at 21 months	Outpatient follow‐up at 12 months	Outpatient follow‐up at 15 months

Abbreviations: bpm, beats per minute; C/S, c‐section; DOL, day of life; ECG, electrocardiogram; EGA, estimated gestational age; fMCG, fetal magnetocardiogram; ICD, implantable cardiac defibrillator; LQTS, long QT syndrome; NSR, normal sinus rhythm; SVD, spontaneous vaginal delivery, TdP, torsades de pointes; VUS, variant of unknown significance.

### 2.1. Case 1

**Figure 1:**
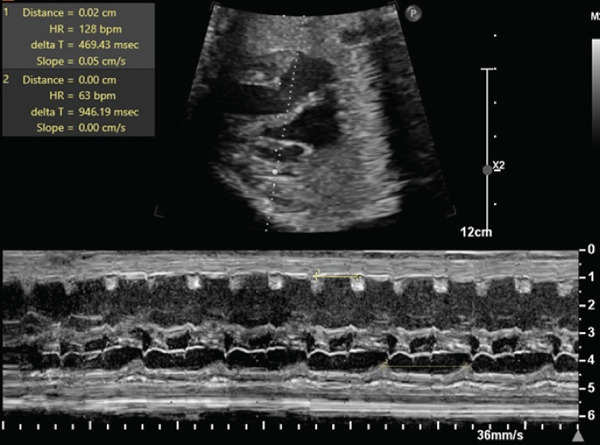
Fetal echocardiogram M‐mode image for Case 1 depicting 2:1 AV block with ventricular rate 63 bpm and atrial rate 128 bpm.

A 32‐year‐old G2P1 presented for fetal echocardiogram at 29.6 weeks gestation due to reported abnormal fetal heart rhythm. She had a known history of LQTS Type 2 diagnosed after an episode of torsades de pointes (TdP). Subsequent genetic testing revealed *KCNH2* variant *c*.1935*G* > *A* *(p.Met645Ile)*, and she is now status post‐ICD and on nadolol therapy. She also had a previous child with LQTS and there are multiple other relatives with the same diagnosis. Initial fetal echocardiogram demonstrated fetal bradycardia with 2:1 AV block (Figure 1). A fetal magnetocardiogram (fMCG) obtained demonstrated QTc of 550 ms and occasional 2: 1 AV conduction. Vitamin D and magnesium supplementation were added to the prenatal treatment regimen, and biweekly fetal echocardiography monitoring continued for the remainder of gestation. A 3.99‐kg male infant was delivered by c‐section at 38.2 weeks gestation. Initial ECG demonstrated normal sinus rhythm with markedly prolonged QTc at 574 ms. Propranolol started on day of life (DOL) 1 with IV lidocaine and mexiletine added on DOL 3 due to nonsustained ventricular tachycardia (VT). Dual chamber epicardial pacemaker was placed on DOL 10 due to continued intermittent bradycardia and insufficient cardiac output. The infant was 36 months old at last outpatient follow‐up and doing well.

### 2.2. Case 2

A 29‐year‐old G2P1 presented for fetal echocardiogram at 32.4 weeks gestation due to concern for fetal arrhythmia. There was no known family history of LQTS, significant arrhythmia, or sudden unexplained death. Initial fetal echocardiogram demonstrated variable high‐degree AV block, abnormal appearing myocardium, and severe biventricular systolic dysfunction. A fMCG demonstrated significantly prolonged QTc over 600 ms, periods of TdP at 200 bpm, and otherwise predominantly 2:1 and 3: 1 AV block (Figure 2). The patient was urgently admitted to initiate maternal oral administration of antiarrhythmic therapy with propranolol and magnesium supplementation to provide transplacental therapy to the fetus. On therapy, the fetus converted to normal sinus rhythm with normalization of ventricular function and was monitored for the remainder of gestation. Parental ECGs and subsequent genetic testing were normal. There was an uncomplicated spontaneous vaginal delivery (SVD) at 38.6 weeks gestation of a 3.2‐kg male infant. Initial ECG demonstrated sinus bradycardia with markedly prolonged QTc at 605 ms. Neonatal propranolol therapy was started on DOL 1 and genetic testing was obtained which resulted in de novo *KCNH2* variant, *c*.1898*A* > *G* *(p.Asn633Ser)*. Mexiletine therapy was then added prior to hospital discharge. Subsequent ECG at 1 year old demonstrated normal sinus rhythm with prolonged QTc of 488 ms and abnormal T‐wave morphology. The infant was 31 months old and doing well at the most recent outpatient follow‐up appointment.

Figure 2fMCG for Case 2 depicting prolonged QTc of 616 ms from fetal averages for Run 1 (a) and episode of TdP noted on Line 3 during Run 1 (b).

**Figure:**
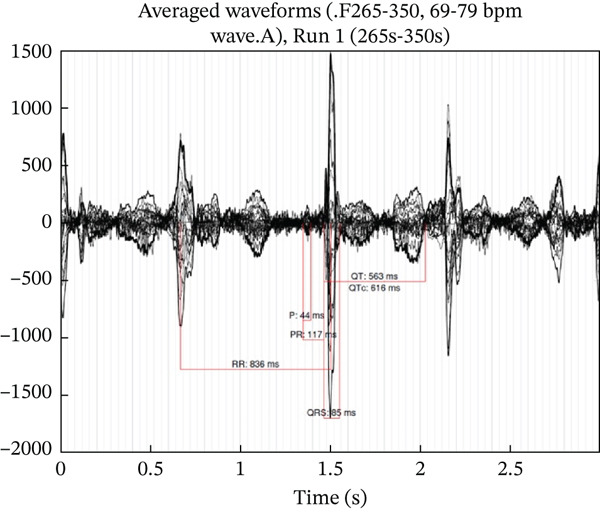
(a)

**Figure:**
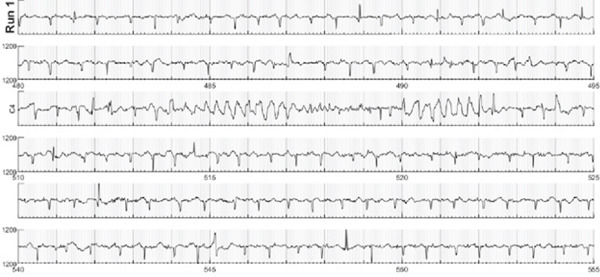
(b)

### 2.3. Case 3

A 34‐year‐old G1P0 was referred for fetal echocardiography due to concern for aortic arch abnormality at 30.2 weeks gestation. There was no known family history of LQTS, significant arrhythmia, or sudden unexplained death. Initial fetal echocardiogram demonstrated fetal bradycardia with fetal heart rates of 115–124 bpm, which are below the third percentile for gestational age, and no other structural abnormalities, concerning for LQTS. A fMCG demonstrated bradycardia with prolonged QTc of 517 ms. Maternal ECG demonstrated borderline or prolonged QTc (457–554 ms), and she was followed biweekly with fetal echocardiograms for the remainder of pregnancy without needing any treatment. There was an uncomplicated SVD at 37.1 weeks gestation of a 2.7‐kg male infant. Initial ECG demonstrated sinus bradycardia with a prolonged QTc of 503 ms and propranolol therapy was started. Subsequent neonatal and maternal genetic evaluation revealed two variants of unknown significance (VUS) in *CALM2* *c*.272*G* > *T* *(p.Arg91Leu)* and *AKAP9 c.10273_10275del (p.Gln3425del)*. Follow‐up ECG at 6 months of age demonstrated normal sinus rhythm with prolonged QTc at 477 ms. The infant was 21 months old at the most recent outpatient follow‐up appointment and doing well.

### 2.4. Case 4

A 32‐year‐old G2P0 was referred for fetal echocardiography for fetal bradycardia at 21.1 weeks gestation. Her paternal aunt had known LQTS with reported *KCNQ1* variant, and her father had a prolonged QTc on ECG but no known genetic testing. She had one sibling who died suddenly in their 30s, and she has a history of syncope with reportedly normal cardiac workup. Initial fetal echocardiogram demonstrated fetal bradycardia with rates of 119–126 bpm. Maternal genetic testing during pregnancy was positive for pathogenic variant in *KCNQ1* *c*.1615 > *T* *(p.Arg539Trp)*, and she was started on propranolol. She was followed with monthly fetal echocardiograms for the remainder of pregnancy and fMCG revealed similar bradycardia with prolonged QTc of 536 ms at 32.2 weeks gestation. There was an uncomplicated SVD at 39.3 weeks gestation of a 2.9‐kg male infant. Initial ECG demonstrated T‐wave abnormality and prolonged QTc at 561 ms. Postnatal genetic testing confirmed the same *KCNQ1* *c*.1615*c* > *T* *(p.Arg 539Trp)* variant. He was started on propranolol and subsequent ECG at 6 months old continued to show sinus bradycardia with prolongation of QTc at 489 ms. The infant was 12 months old and doing well at the most recent outpatient follow‐up.

### 2.5. Case 5

A 36‐year‐old G4P1 was referred for fetal echocardiogram at 21.6 weeks gestation due to known history of maternal LQTS and *KCNQ1* *c*.914*G* > *A* *(pTrp305* ∗) variant. The patient was on propranolol therapy. Her father, two sisters, and four of six nieces and nephews also have known LQTS with the same variant. Initial fetal echocardiogram demonstrated mild sinus bradycardia with rates of 127–138 bpm. Fetal genetic testing was completed and again consistent with the same *KCNQ1* *c*.914*G* > *A* *(pTrp305* ∗) variant and she was monitored monthly with fetal echocardiograms for the remainder of gestation. There was an uncomplicated SVD at 39.2 weeks gestation of a 3.7‐kg male infant. Initial ECG demonstrated sinus bradycardia with prolonged QTc at 545 ms with abnormal T‐wave morphology and propranolol therapy was started. Follow‐up ECG at 3 months of life revealed normal sinus rhythm with prolonged QTc of 461 ms and continued abnormal T‐wave morphology. The infant was 15 months old at last outpatient follow‐up and doing well on therapy.

## 3. Discussion

LQTS is a cardiac ion channelopathy characterized by prolonged ventricular repolarization with risk for life‐threatening ventricular arrhythmias including TdP and VT [1]. The prolonged ventricular repolarization presents as QT prolongation and T‐wave abnormalities on ECG in patients of any age, however the clinical presentation ranges widely from asymptomatic to cardiac death [2]. The variability is affected by genetic etiology, QT duration, and treatment adherence [3]. With appropriate identification and monitoring, risk stratification, and treatment, this risk has decreased significantly in recent years with < 1% mortality risk [4].

The true prevalence of congenital LQTS is challenging to determine; some affected fetuses may experience in utero fetal demise from ventricular arrhythmias before the condition is recognized [5]. Historical reports indicated population prevalence of 1: 5000 to 1: 20,000; however, newer studies indicate it may be as high as 1 in 2000 [6]. Families with LQTS have increased chances of poor outcomes during pregnancy with miscarriage risk doubled and stillbirth risk eight times higher than the general population [7].

### 3.1. Fetal Diagnosis of LQTS

The most common tool for prenatal cardiac evaluation is fetal echocardiography; however it cannot perform a direct measurement of fetal QT interval. Therefore, the primary mode of prenatal diagnosis of LQTS is identification of a detectable hallmark fetal rhythm such as fetal bradycardia, 2:1 AV block, or ventricular arrhythmias [8]. Cases 1 and 2 demonstrated these with 2:1 AV block and ventricular arrhythmia, respectively (Figures 1 and 2). Left ventricular isovolumic relaxation time (IRT) by fetal echocardiography can also be a marker suspicious for fetal LQTS, and Phan et al. recently linked the fetal QT by fMCG to the IRT by echo Doppler, providing another means of recognizing the severity of the QT lengthening [9, 10].

Historically, fetal bradycardia was defined as a fetal heart rate less than 110 bpm irrespective of the gestational age [11]. However, this unified fetal heart rate cutoff has lower sensitivity to detect significant fetal bradycardia given that average fetal heart rate normally decreases throughout gestation. More recently, repeated fetal heart rates less than the third percentile for gestational age has been proposed as a more sensitive marker for fetuses requiring screening for possible LQTS [12, 13]. Cases 3, 4, and 5 in our series were initially referred due to fetal bradycardia identified using this cutoff of the third percentile for gestational age but would have been missed using a cutoff of 110 bpm. In contrast, Case 2 was referred for evaluation due to significant arrhythmia in late gestation despite fetal heart rates < 3*%* documented from ~18 weeks gestation.

Newer investigational technologies such as fMCG have provided evaluation of actual electrical signals in the fetus allowing more accurate identification of fetal patients with potential LQTS [14]. With accurate identification of LQTS and high sensitivity in detecting TdP episodes, fMCG identified cases not recognized by fetal echocardiography, similar to Case 2 in our series (Figure 2) [15]. The Heart Rhythm Society′s 2023 consensus statement on arrhythmias in pregnancy gave fMCG a Class 2a designation for evaluation of known or suspected inherited arrhythmia syndrome cases [16]. Unfortunately, fMCG is not broadly available currently.

### 3.2. Genetics in Diagnosis of LQTS

Genetic testing is crucial for diagnosis in LQTS [17]. With clear genotype–phenotype associations for the most common forms of LQTS, genetic testing can confirm the clinical diagnosis, guide management, and provide prognostic information [18]. As understanding of genetic contributors to LQTS evolves, including identification of rare presentations and syndromes with overlapping features, genetic testing is increasingly useful for risk stratification and pharmacotherapy choices [19].

Our cases represent both classic and atypical fetal LQTS. Cases 4 and 5 represent the most well understood form of LQTS, Type 1, due to pathogenic variants in *KCNQ1*. LQTS Type 2 with variants in *KCNH2* is also a common type of LQTS, making the diagnosis in cases 1 and 2 consistent with published literature [13].

Evaluation of cases without a known family history poses a challenge for clinical teams. Classification of the *KCNH2* *c*.1898*A* > *G* *(p.Asn633Ser)* variant for the fetus in Case 2 was straight forward despite being de novo in the fetus. Cases with de novo variants for LQTS tend to be more malignant with longer QTc intervals and higher risk of VT/TdP, functional 2:1 AV block, and death [20]. The documentation of TdP on fMCG for Case 2 is consistent with this trend.

In contrast, the *KCNH2* *c*.1935*G* > *A* *(p.Met645Ile)* variant identified in Case 1 was classified as a VUS in the mother′s previous pregnancy. Since a VUS cannot be used for clinical decision‐making, our cardiovascular genetics team conducted systematic cascade family screening in a sufficient number of affected family members to reclassify the variant as likely pathogenic. As noted by Leung et al., it is increasingly important to reclassify VUS to better inform our ongoing genetic knowledge of LQTS [21].

The fetus in Case 3 was identified to have a VUS in *CALM2,* *c*.272*G* > *T* *(p.Arg91Leu)*, determined to be maternally inherited. *CALM2*‐related arrhythmia is a newly recognized disorder, and the full fetal clinical spectrum is yet to be characterized; however, sudden cardiac death and stillbirth have been reported [22]. Our cases involving clinical and family history correlation for VUS highlight the important role that clinicians play in tailoring care for patients while improving understanding of the genetic underpinnings of fetal arrhythmia.

### 3.3. Multidisciplinary Approach to Prenatal Management

For fetal patients with prenatal testing suspicious for LQTS, a multidisciplinary approach is critical. The team should include fetal cardiology, genetic counseling, pediatric electrophysiology, adult electrophysiology or cardiology, MFM, and neonatology [16, 23–25]. Communication between team members is key to ensure prompt diagnosis and treatment to decrease the risk of in‐utero complications or fetal demise and also plan safe maternal care should testing uncover previously undetected maternal LQTS. For Case 1, collaboration with the pediatric cardiovascular genetics team allowed for full evaluation and understanding of the family′s genetic etiology for LQTS. Interdisciplinary involvement of the fMCG medical physics team also provided early recognition of the prolonged QT interval. In Case 2, multidisciplinary communication was key to allow immediate admission to the MFM inpatient team after diagnosis of TdP on fMCG for initiation of magnesium and propranolol therapy, thereby decreasing the risk of in utero fetal demise.

In any case of suspected fetal LQTS, incorporating genetic counseling to discuss testing options and results provides additional crucial information for pregnancy and delivery management. In all our cases, patients received genetic counseling with a certified genetic counselor at the time of diagnosis. Risks and benefits of prenatal genetic testing options were discussed in detail. In four of five cases, patients chose to defer genetic testing until after delivery because the benefits of prenatal genetic testing did not outweigh the risks of amniocentesis. In Cases 1, 4, and 5, a known variant in the family clarified expectations for the fetus, delivery planning, and neonate. In Cases 2 and 3, the fetal phenotype appeared relatively mild or clinical diagnosis was made late enough in gestation that the risk of preterm delivery after amniocentesis was deemed not worth the benefit of definitive genetic diagnosis.

MFM specialists play an important role for these mother–fetus dyads. In cases of fetal TdP, prompt recognition and treatment with magnesium and propranolol can prevent intrauterine fetal demise [14]. Any maternal therapy requires close monitoring of potential side effects, such as maternal cardiac rhythm disturbances due to the antiarrhythmia treatment or fetal growth restriction due to maternal beta‐blocker therapy [24]. The MFM team is also heavily involved in delivery planning for these patients. Although most can undergo vaginal delivery, modifications may be needed based on fetal rhythm status. If the mother also has LQTS, QT‐prolonging anesthetics and other medications should be avoided [24].

As a part of the multidisciplinary care team, pediatric cardiologists, electrophysiologists, surgeons, and cardiac ICU providers can provide additional counseling on immediate postnatal care expectations and long‐term management strategies. Prenatal parental counseling on the potential need for neonatal pacemaker insertion aids in parental preparedness prior to birth. The counseling can include techniques of neonatal pacemaker insertion, gestational age and weight cutoffs for various approaches, and temporary options for low‐birthweight neonates. The prenatal period can be an ideal time to identify CPR knowledge among those who will be caregivers for the infant and begin training at a less stressful time.

## 4. Conclusion

LQTS diagnosis in fetal patients is challenging, but it is essential in order to initiate appropriate medical therapies to decrease the likelihood of sudden cardiac death in both prenatal and postnatal settings. Genetic evaluation plays a key role in making or confirming the diagnosis in these patients. Our institutional standard follows the recommended multidisciplinary team approach with regular team meetings to aid in patient, family, and care team preparedness for delivery. This approach also includes multispecialty care coordination and counseling to educate patients regarding pregnancy management, potential medical therapies, and delivery planning allowing optimization of outcomes for both the fetus and mother [26].

## Funding

No funding was utilized for this case series. Two authors would like to acknowledge unrelated grant support: J.F.S. and R.T.W.—NIH R44HL160257; J.F.S.—NIH R01HL143485.

## Consent

No written consent has been obtained from the patients as there is no patient identifiable data included in this case report.

## Conflicts of Interest

The authors declare no conflicts of interest.

## Data Availability

Data sharing is not applicable to this article as no datasets were generated or analyzed during the current study.
